# Promoting Prevention Through the Affordable Care Act: Workplace Wellness

**DOI:** 10.5888/pcd9.120092

**Published:** 2012-12-13

**Authors:** Laura Anderko, Jason S. Roffenbender, Ron Z. Goetzel, Francois Millard, Kevin Wildenhaus, Charles DeSantis, William Novelli

**Affiliations:** **Author Affiliations:** Jason S. Roffenbender, Charles DeSantis, William Novelli, Georgetown University, Washington, DC; Ron Z. Goetzel, Truven Health Analytics, Atlanta, Georgia; Francois Millard, Vitality Group, Chicago, Illinois; Kevin Wildenhaus, Johnson & Johnson, Wellness and Prevention, Inc, Fort Washington, Pennsylvania.

## Abstract

Public health in the United States can be improved by building workplace “cultures of health” that support healthy lifestyles. The Affordable Care Act (ACA), which includes the Prevention and Public Health Fund, will support a new focus on prevention and wellness, offering opportunities to strengthen the public’s health through workplace wellness initiatives. This article describes the opportunity the ACA provides to improve worker wellness.

## Background

Declining workforce health contributes to an increase in health-related expenses, both in direct medical payments and indirect costs resulting from absenteeism and presenteeism (1–4). Wellness programs have been shown to save money; however, such programs are underused (3,4). One reason may be that the future benefits of healthy employees are significantly undervalued relative to the cost. Despite this, many businesses are taking a holistic approach to health by offering wellness programs to their employees.

According to a survey of large manufacturing employers, 77% offered some form of wellness programs (5). Employers with fewer than 500 employees offer wellness programs at a lower rate than employers with more employees, although a recent survey found that 29% of small businesses offered some kind of wellness options compared with 16% five years ago (5,6).

With the passage of the Affordable Care Act (ACA), worksite wellness programs will become part of a national public health strategy to address the increase in chronic diseases, which are predicted to cost the US health care system an estimated $4.2 trillion annually by 2023 (7). Evidence suggests that worksite wellness programs are cost-beneficial, saving companies money in health-care expenditures and producing a positive return on investment (ROI). Baicker et al calculated an average return of $3.27 in medical costs for every dollar spent on worksite wellness programs (5). Organizations that have reported cost savings or positive ROI ratios include Johnson & Johnson, Citibank, Procter & Gamble, Chevron, California Public Retirement System, Bank of America, DuPont, Duke University, and Highmark (1,8).

The Prevention and Public Health Fund (PPHF) of the ACA contains many new provisions designed to improve public health and wellness. The ACA was designed to address 4 key prevention areas: 1) community prevention, 2) clinical prevention, 3) public health infrastructure and training, and 4) research and surveillance focused on workforce wellness. Understanding key issues that affect the American workforce is critical to improving prevention efforts (9).

## Workforce Trends and Wellness

The health of the modern workforce is changing. The aging of the labor force contributes to the complexity of health needs; chronic disease has become a significant concern for employers, due to escalating health care costs. The group of workers aged 55 or older, which comprised 13% of the labor force in 2000, is projected to increase to 20% by 2020 and account for 19% of the labor force by 2050 (1,10). Employees are older, and the numbers of employees with chronic conditions, including depression, anxiety, and diabetes, are increasing (11). Many have multiple chronic conditions, complicating medical management and making wellness interventions potentially more valuable because they affect multiple conditions at once.

Workforce conditions are also changing. The decrease in acute traumatic injuries from work and the increase in chronic conditions such as depression and anxiety reinforce the need to improve the availability of wellness programs (1). Depression and stress are major sources of lost productivity in the workplace (11). The World Health Organization estimates that depression will be the most prominent disability at work in the next decade (1). Self-reported data from more than 1.3 million employees indicate that 30% experienced some depression or were in treatment (1,11,12). Depression symptoms affect productivity and work time, and many people with depression may have other health problems (1,12).

The inclusion of workplace wellness program funding in the ACA increases the potential for these programs to improve the declining health of an aging demographic. Through the social and organizational support structures of the workplace, wellness programs can be integrated effectively into the lives of a demographically shifting workforce that is steadily growing less healthy. Because more than 60% of Americans obtain their health insurance coverage through an employment-based plan, employee wellness programs are uniquely positioned to respond to the varied health needs of a multigenerational workforce (13).

## The Workplace as a Microcosm of Society

The workplace, as a microcosm of society, has the potential to improve health substantially in the United States by building a culture of health that facilitates healthy lifestyles for employees. This culture can be created when the employer provides 1) financial and organizational support for evidence-based health promotion interventions; 2) consistent communication with workers that encourages positive health behaviors; 3) social and organizational supports from peers and supervisors; 4) policies, procedures, practices, and organizational norms that support a healthy lifestyle (for example, access to healthy foods and physical activity or banning smoking on company grounds); 5) financial or other types of incentives for participation in health improvement activities; and 6) a common purpose that is dedicated to a healthier workforce. The workplace also has the advantage of reaching large segments of the population not exposed to or engaged in organized health improvement efforts (1).

In companies with a strong culture of health, employees are 3 times as likely as others to report taking action to improve their health (14). These same employees rate all aspects of their performance higher than employees whose employers lack a strong culture of health. An employer’s commitment to employee well-being is as critical to overall job satisfaction as opportunity for advancement and more important than competitiveness of pay and benefits (1). Additionally, companies with a strong culture of health have better financial outcomes and lower employee turnover (14). However, fewer than 26% of employees believe their company has a strong culture of health (1).

## Worker Wellness and the ACA

The PPHF represents the largest national commitment to investing in wellness and prevention. Its 3 major prevention provisions are waiving cost sharing for preventive services, providing new funding for community preventive services, and creating workplace wellness programs (15). The Centers for Disease Control and Prevention will study these programs and determine which elements produce the best results (15).

Although the US Department of Health and Human Services distributed initial worksite wellness plan grants in 2011 ($10 million in ACA funds were released for the creation of workplace wellness programs) (15,16), future funding is in jeopardy. New legislation passed by Congress in February 2012 (HR 3630, the Middle Class Tax Relief and Job Creation Act) reduces the fund’s spending by $5 billion over 10 years from the initial $15 billion, starting in fiscal year 2013 (15). Some in public health worry that funds will be used to offset reductions in existing federal health programs rather than to support new prevention efforts (15). In July 2012, the Supreme Court upheld the ACA with public health provisions intact. Although funding has not yet been made available, the PPHF includes $200 million for small business wellness plan grants and is a major component of health reform (17).

## Bridging Work and Wellness: Lessons Learned

Modifiable health risks that lead to disease can be decreased through workplace-sponsored health promotion and disease prevention programs. The importance of the worksite as a means for promoting health is underscored by its inclusion in Healthy People 2020 (18). To be successful, wellness programs must be comprehensive, tailored to the population, creatively marketed, and embraced by top management (1).

When growth in worker wellness programs is supported through the ACA, programs must be developed by using an evidence-based prevention framework for preventive health (1). An effective worker wellness program incorporates essential components such as the organization’s culture and leadership, program design, program implementation and resources, and program evaluation. Evaluation criteria can include financial data (eg, cost savings), health outcomes (eg, risk reduction), quality of life, and productivity (1).

## Nontraditional Settings for Worksite Wellness

In addition to traditional worksite wellness settings, wellness programs have been developed in nontraditional settings such as universities. These programs have resulted in outcomes similar to those seen in industry, providing faculty and staff and their dependents with access to wellness resources and programs, integrating a wellness philosophy across campus, promoting preventive health care, and empowering people to become responsible for their physical and emotional health. Programs may include wellness activities such as screenings or walking clubs, which have the capacity to create a sense of community, improve morale and productivity, reduce employee turnover, reduce absenteeism, and contain health care costs (1,13,19). Lessons learned from wellness programs offered at academic centers can inform future community-based workplace wellness endeavors.

Academic exemplars include Georgetown University and the University of Miami. Each university has established a wellness philosophy that outlines a framework and expectations for promoting health with an emphasis on community benefits. Both emphasize a commitment to promoting wellness through programs such as stress management, weight reduction, yoga, smoking cessation, and walking clubs. A few years ago, Georgetown established the “Weekly Walk to the Mall,” a 4-mile hike to the National Mall for employees and community residents (http://wellness.georgetown.edu). Recently, nearly 300 employees from 3 campuses at the University of Miami participated in a walking competition during a 12-week program, exceeding 10,000 steps per day. Winners earned round-trip airline tickets (20).

## Effectiveness of Incentives

A systematic literature review of 47 randomized controlled trials found that economic incentives worked an average of 73% of the time (21). Traditional economic incentives, which use simple, one-time approaches such as providing cash, gift cards, or health plan benefit discounts, are effective in the short run for simple preventive care, short-term health behavior changes (eg, seeking a health risk assessment), and distinct, well-defined behavioral goals (eg, immunizations). Small incentives can produce finite changes, but it is not clear what size incentive is needed to yield a sustained effect (eg, weight control). The use of financial incentives is likely to be more effective as part of a combination prevention approach for long-term behavior change (1,22). More effective approaches offer incentives that workers would not otherwise have, such as access to affordable, healthful food. These incentives increase the likelihood that workers will repeat the healthy behavior (22).

Another consideration is the cost versus the value of the incentive. For example, providing a $100 incentive is a cost; providing a discount for something healthy is a value. Offering incentives through the accumulation of points toward travel (free flights), movies, music downloads, and retail discounts, similar to rewards programs offered by airline and credit card companies, has been effective in improving health. Workers can redeem points for items of perceived value that can have a low absolute cost but have high value to the individual (1,22).

Another innovative strategy that is gaining in popularity includes using lottery incentives to change health behavior. Lottery incentives can provide frequent small payoffs (eg, a 1 in 5 chance daily for a $10 award if the employee’s 2-digit number is randomly selected and weight reduction goals are met). The evidence suggests that people are attracted to them and they can help people reach health behavior goals, including weight loss and maintenance. For example, in 1 study, obese volunteers were divided into 3 groups: 1 had no financial incentives to lose weight; 1 was paid a monthly, contracted “fee” based on how much weight members lost; another was enrolled in a lottery program providing up to $100 a day for losing weight. The participants of the contract and lottery groups lost significantly more weight than members of the comparison group after the 16-week period (23).

Finally, taxing unhealthful foods through policy initiatives while providing discounts or incentives on healthy purchases through business initiatives have been effective in changing health behaviors internationally (1). Although limited in scope, efforts to change behavior through taxation on unhealthful foods have had promising results in the United States. One study found that a 35% tax on sugar-sweetened drinks ($0.45 per drink) led to a 26% decline in sales and concluded that a 20% tax on these drinks would reduce obesity levels by 3.5% in US adults (24).

## Improving Health Through the Power of Community

The health of the individual is inseparable from the health of the community, and the health of the community is inseparable from the health of the nation. Community engagement to promote health is good business. Community interventions can support worksite programs and policies and make it easier for employees and their families to make healthy choices, especially for hard-to-reach populations (1,25). Employers can support community and population health initiatives by taking the following steps:

- Promoting best practices in health promotion.
- Sharing planning, implementation, and marketing expertise to target initiatives.
- Engaging in community awareness and education.
- Supporting smoke-free and other policies, such as bike and walking paths, within community locations (1).

Innovative partnerships between public health and community-based organizations and businesses at the community level are changing workplace wellness, resulting in a growing number of community-based workplace wellness programs. One example includes the partnership between the Johnson & Johnson Health Care Institute and the University of California, Los Angeles, which provided materials and training to Head Start parents to enhance health literacy related to diabetes prevention and nutrition. In Detroit, DTE Energy developed a Gardens Project that enlisted help from schools, community, and religious groups; project gardens yielded more than 44,000 pounds of fresh produce (1). Small businesses will benefit from programs such as these that are scalable and implemented incrementally. In an effort to improve the health of patients and employees and to benefit the local community, some hospitals in partnership with the American Heart Association have begun offering farmer’s markets and gardens on hospital grounds to increase physical activity and promote more healthful eating (http://healthierhospitals.org/).

## Conclusion

Real health reform starts with prevention. Focusing on improving the health and quality of people’s lives will improve the productivity and competitiveness of our workers and citizens (1). The ACA provides our nation with an opportunity to broaden the scope of worker wellness by expanding our efforts to include more workers and their communities. Although most programs under ACA are funded through mandatory rather than discretionary spending, funding is in jeopardy because of competing economic priorities. It is critical to the health of our nation that the provisions in the ACA for employee wellness remain a priority.

Beyond the opportunities presented by the ACA to improve the nation’s health through worker wellness is the need to focus on prevention and public health and to emphasize sociopolitical strategies to improve health (eg, offering discounts on healthful foods). Combining healthy public policy with worker wellness initiatives could result in significant improvements in not only our nation’s health but also our economy.
